# Localized Proteasomal Degradation: From the Nucleus to Cell Periphery

**DOI:** 10.3390/biom12020229

**Published:** 2022-01-29

**Authors:** Xing Guo

**Affiliations:** 1 Life Sciences Institute, Zhejiang University, Hangzhou 310058, China; xguo@zju.edu.cn; 2 Zhejiang Provincial Key Laboratory for Cancer Molecular Cell Biology, Hangzhou 310058, China

**Keywords:** proteasome, membrane, nucleus, condensate, ubiquitination, myristoylation

## Abstract

The proteasome is responsible for selective degradation of most cellular proteins. Abundantly present in the cell, proteasomes not only diffuse in the cytoplasm and the nucleus but also associate with the chromatin, cytoskeleton, various membranes and membraneless organelles/condensates. How and why the proteasome gets to these specific subcellular compartments remains poorly understood, although increasing evidence supports the hypothesis that intracellular localization may have profound impacts on the activity, substrate accessibility and stability/integrity of the proteasome. In this short review, I summarize recent advances on the functions, regulations and targeting mechanisms of proteasomes, especially those localized to the nuclear condensates and membrane structures of the cell, and I discuss the biological significance thereof in mediating compartmentalized protein degradation.

## 1. Introduction

The 26S proteasome is situated at the core of the ubiquitin–proteasome system (UPS), responsible for selective degradation of the majority of cellular proteins in eukaryotes. For over three decades since its discovery, the proteasome has been thoroughly studied with regard to its composition, structure, activity, regulation and relation to health and disease. The fully assembled 26S proteasome holoenzyme consists of a 20S core particle (CP, formed by homologous α and β type subunits) and one or two 19S regulatory particles (RP, formed by six ATPase subunits called Rpt1-6, and thirteen non-ATPase subunits known as Rpns) [1,2,3,4,5,6,7,8,9,10]. Recent structural studies have significantly furthered our knowledge about how the proteasome recognizes and processes ubiquitinated substrates [6,11,12]. The success of proteasome inhibitors (e.g., Bortezomib/Velcade^®^) in treating multiple myeloma [13] has spurred intensive research on developing proteasome-targeting compounds for therapeutic uses toward cancer and autoimmune diseases, whereas (re-)activating the proteasome by small molecules has also emerged as an attractive strategy for alleviating symptoms associated with neurodegeneration and aging [14,15,16,17,18,19]. A better understanding of the function and regulation of the proteasome is of great biological and clinical importance.

As a soluble and highly abundant macromolecular complex [20,21,22], the proteasome resides in both the nucleus and cytoplasm of a cell and has been found associated with various subcellular structures, including the chromatin, cytoskeleton, nuclear envelope, plasma membrane, the cytosolic side of membrane-bound organelles and membraneless organelles/condensates (see below). Despite their pervasive presence, proteasomes are not evenly distributed in all cells. On a global scale, asymmetric cell division can lead to unequal inheritance of proteasomes between the daughter cells [23,24,25,26]. The specific subcellular localizations of proteasomes are often cell type- and growth status-dependent and dynamically regulated under both basal and stimulated/stress conditions [27,28,29]. A classic example is that in yeast, proteasomes are predominantly present in the nucleus of proliferating cells; but upon quiescence or carbon starvation, nuclear proteasomes are rapidly exported to the cytosol, where they are concentrated in a membraneless structure called proteasome storage granule (PSG) [30]. PSGs quickly resolve when yeast cells resume growth in nutrient-rich media and proteasomes re-gather in the nucleus. This reversible process is believed to protect the proteasome repertoire from autophagic degradation under stress conditions, while allowing them to regain function as soon as the stress is relieved [30,31].

Proteasomes also exist extracellularly. Original studies have shown that secreted proteasomes from ascidian sperms can digest vitelline coat proteins outside the egg and are required for egg penetration and fertilization [32,33,34]. Circulating proteasomes (c-proteasomes) were also found in humans around the same time [35], which has been confirmed by a series of subsequent studies (see reviews [36,37,38] and references therein). Present in the blood as well as other bodily fluids, these c-proteasomes are mostly in the form of 20S, probably due to the low-ATP extracellular environment that does not support RP–CP association [39,40]. Nonetheless, they are enzymatically active, and elevation of their levels is often correlated with either malignancy or tissue injury/damage, making them a promising biomarker for disease diagnosis [36,37,38]. How the proteasomes exit the cell remains a matter of debate, although a likely mechanism is via exosome-mediated non-conventional secretion [36,41]. The pathophysiological roles and regulatory mechanisms of extracellular proteasomes have yet to be fully understood.

Various mechanisms have been identified to target protein substrates to different subcellular regions for proteasomal degradation [42,43,44,45,46,47,48,49,50]. On the flip side, proteasomes should be available at the site of degradation or can be mobilized to meet the substrates. In addition to the examples introduced above, the dynamic localizations of the proteasome have been extensively studied (particularly in yeast) and summarized in a series of reviews [51,52,53,54,55]. Here, I will focus on the latest findings about nuclear-localized and membrane-associated proteasomes in mammalian cells and discuss the targeting mechanisms, biological functions, as well as regulations of proteasomes at these specific compartments.

## 2. Proteasomes in the Nucleus

A considerable amount of proteasomes exist in the nucleus, where they play pivotal roles in regulating mitotic cell cycle [56,57], meiosis [58,59,60], transcription/chromatin remodeling/epigenetic control [61,62,63,64], RNA splicing [65,66], DNA damage repair [67,68,69] and nuclear protein quality control [42,49,70], among others. There are also nuclear-specific proteasome activators (e.g., PSME3/REGγ and Blm10) and regulators that control these processes [71,72,73,74,75].

### 2.1. Nuclear Targeting of the Proteasome

In yeast, nuclear proteasomes are essential for cell survival [76]. Unlike in higher organisms, the nuclear envelope (NE) of a yeast cell remains intact during closed mitosis, and active nuclear import of the proteasome across the NE is necessary for continuous cell proliferation. Early studies identified putative nuclear localization signals (NLS) in certain α subunits of yeast and archaeal proteasomes, some of which were later found to be functionally conserved in their mammalian homologs [77,78,79,80,81]. Direct recognition of NLS by the importin complex is therefore important for trafficking proteasome subunits and sub-assemblies into the nucleus [51].

NLS has also been identified in the 19S subunits, Rpn2 and Rpt2. Although both of these NLS sequences are functional in binding importins and guiding non-nuclear proteins to the nucleus in yeast, only the Rpn2-NLS was shown to be primarily responsible for nuclear import of the 19S base complex (including Rpt1-6, Rpn1, 2, 10 and 13). The putative NLS of Rpt2 only provided secondary functions when the Rpn2-NLS was inactivated [82]. In mammals, these NLS sequences are only partly conserved in Rpn2 and Rpt2, and their relevance to nuclear localization of the proteasome has not been examined. Of note, positively charged residues at the C-terminal half of the bipartite ScRpt2-NLS (KFGRKKRK) are also present in human Rpt2 (_32_RVGKKKKK_39_), a region sandwiched between Rpt2 and its neighboring subunit Rpn1, possibly inaccessible to the importins [12]. On the other hand, the N-terminal part of ScRpt2-NLS roughly corresponds to _15_KKDDKDKKKK_24_ of human Rpt2, which was recently suggested to form a charge–charge interaction with phosphorylated Ser361 of Rpn1. This interaction is required for proper proteasome assembly in human cells, and mutation of these lysine residues weakened Rpt2 incorporation into the proteasome [83]. It should be pointed out that Rpn1-Ser361 phosphorylation does not occur in yeast, given the lack of phosphosite, therefore the ScRpt2-NLS may be more available for importin binding. In addition, aa. 1–24 of human Rpt2 is sufficient for bringing GFP to the cell membrane [84] largely due to N-myristoylation of this sequence (see below), while the myristoylation site was omitted in the GFP targeting study with yeast Rpt2-NLS [82]. These observations suggest that similar NLS-like motifs of the same proteasome subunit may have different functions in mammalian cells and yeast, which may be determined by the subtle sequence differences, assembly status and modifications of the subunit itself and its adjacent subunits.

The proteasome may be imported into the nucleus as free subunits and assembly intermediates as noted above, and it can also pass through the nuclear pore complex (NPC) as assembled 20S or 26S particles [21,85]. This is possible owing to the elasticity of the NPC structure [86,87] and depends on the importins. In addition to direct importin binding to the NLS sequences, which may be masked in the proteasome complex [51,77,80], nuclear translocation of the 20S and 26S proteasomes can be facilitated by NLS-containing adaptor proteins that simultaneously interact with the proteasome and importins. Sts1 in budding yeast [88,89,90] and its ortholog, Cut8, in fission yeast [91,92] both possess NLS, form dimers, bind the proteasome and are required for proper localization of proteasomes in the nucleus. A homolog of Sts1/Cut8 has been found in fruit fly. Importantly, both Sts1 and Cut8 are substrates of the proteasome with a very short half-life, and their ubiquitin-dependent (Cut8) and -independent (Sts1) degradation is coupled with their ability to mediate proteasome transport into the nucleus. Thus, these proteins serve as regulators, sensors and targets of nuclear proteasomes.

Very recently, a functional counterpart of Sts1/Cut8 was identified in human cells through a CRISPR screen for regulators of c-myc. Among the top hits was the transcription co-activator AKIRIN2, whose depletion significantly stabilized the c-myc protein, as well as many known nuclear targets of the proteasome [93]. AKIRIN2 is highly conserved through evolution, especially in vertebrates. Showing no obvious sequence identity with Sts1/Cut8, AKIRIN2 functions similarly to these yeast proteins in controlling proteasome nuclear localization. First, AKIRIN2 harbors an N-terminal bipartite NLS recognized by a particular importin, IPO9, which is also important for nuclear import of proteasomes in Drosophila germ cells [94]. Second, AKIRIN2 can homodimerize via its coiled-coil domain and preferentially binds assembled 20S and 26S proteasomes through its highly conserved C-terminal SYVS motif. The cryo-EM structure of the AKIRIN-20S CP complex has revealed that the SYVS tails insert into specific pockets formed between adjacent α subunits that are known to be occupied by the C-termini of Rpt subunits upon RP–CP association. The coiled-coils further lie across the surface of the α ring. This mode of interaction suggests that AKIRIN2 dimers can bind and promote nuclear import of free 20S CP and singly capped 26S proteasome (RP–CP) but not the doubly capped 30S proteasome (RP–CP–RP) [93,95]. Third, same as Sts1 and Cut8, AKIRIN2 is also an unstable protein that can be rapidly degraded by the proteasome. Finally, AKIRIN2 is fundamentally important for nuclear proteasomal degradation and required for cell survival. In particular, following (open) mitosis, AKIRIN2 controls the re-accumulation of nuclear proteasomes after the NE reforms. This fits well with its transcriptional upregulation prior to mitotic onset [93,96]. Together, AKIRIN2 and Sts1/Cut8 constitute a highly conserved strategy used by the cell to ensure a proper abundance of proteasomes in the nucleus.

### 2.2. Nuclear Condensates of the Proteasome

The proteasome is an integral component of the protein quality control system (PQC) [97], which often functions in spatially organized compartments within a cell [48]. Proteasome association with distinct subcellular structures becomes more evident under stress conditions, as typified by the formation of juxtanuclear quality control compartment (JUNQ), insoluble protein deposit (IPOD)/aggresome, intranuclear quality control compartment (INQ), stress granule (SG) and other cellular bodies/aggregates. Much of this knowledge was gained from studies in yeast, while recent findings in higher organisms have highlighted the evolutionary conservation and significance of proteasome compartmentalization in response to proteostasis stress [44,45,46,47,48,50,98,99,100,101,102,103,104,105].

Several groups have recently reported proteasome-containing condensates forming in the nucleus of mammalian cells [106,107,108,109]. Although detected in different cell types with different stimuli, these subnuclear structures share several common features: (1) They quickly appear under stress and then resolve either spontaneously or after the cells are returned to normal conditions; (2) These proteasome foci are distinct from known nuclear structures, such as PML bodies, Cajal bodies, DNA damage foci, nuclear speckles and nucleoli; (3) They are fluid membraneless organelles formed by liquid–liquid phase separation (LLPS) [110]; (4) All of them contain proteins modified by K48-linked ubiquitin chains, which serve as a critical nucleating factor to recruit proteasomes. Specifically, the ubiquitin shuttle protein Rad23B can establish multivalent binding with ubiquitin chains via its tandem ubiquitin-associated (UBA) domains, providing a driving force for LLPS. Rad23B also docks onto the proteasome via its ubiquitin-like (UBL) domain, thus attracting proteasomes to the ubiquitinated proteins [106,109]. Similarly, p62/SQSTM1, a key regulator of macroautophagy, also contains a UBA domain for ubiquitin binding and uses its PB1 domain for proteasome interaction. Nuclear retention of p62 was shown to be sufficient to induce proteasome phase separation [108]; (5) Proteasomes in these nuclear condensates are fully assembled, active 26S complexes, and their proteolytic activity can be enhanced by the high local concentrations of both the proteasome and the substrates. Therefore, in contrast to the cytosolic PSG observed in yeast, which contains dissociated RPs and CPs, stress-induced nuclear proteasome foci in mammalian cells are active degradation centers of ubiquitinated proteins.

There were also unique findings reported about each type of these nuclear proteasome condensates. By treating cells with sucrose, glucose or NaCl, Yasuda et al. first described hyperosmotic stress-induced proteasome phase separation in the nucleus [106]. This process not only relied on ubiquitination (as determined by E1 inhibition) and Rad23B but also on UBE3A, a proteasome-associated E3 ubiquitin ligase that was also present in the condensates. Song et al. later found that, in addition to K48-linked ubiquitin chains, proteins in the same kind of liquid droplets were also modified by K11/K48-branched chains [111]. UCH37, a long-recognized proteasome-associated deubiquitinase (DUB), was also present at the sucrose-induced proteasome foci. With a unique de-branching activity toward K11/K48 and K6/K48 ubiquitin chains [111,112], UCH37, together with the ATPase p97/VCP, played an important role in disentangling the ubiquitinated proteins and resolving the condensates through proteasomal degradation [106,111]. Hyperosmolarity led to nucleolar stress and impaired ribosome biogenesis. Unassembled orphan ribosomal proteins were targeted to the proteasome condensates where they were degraded, thereby preventing their aggregation in the nucleus under stress. As such, the nuclear proteasome droplets serve as an important quality control mechanism to protect the cell from hyperosmotic stress.

Nuclear puncta of proteasomes under hypertonic treatment were also observed by Lee et al. [107]. Using a higher NaCl concentration and prolonged treatment, these investigators noted that proteasome foci decorated the nuclear membrane, probably representing a late-stage response to more severe hyperosmotic stress. The formation of these intranuclear foci was not regulated by the kinase p38, which has been shown to phosphorylate Rpn2 in response to hyperosmotic stimulation [113]. However, pharmacologic inhibition of either importin or exportin abrogated nuclear proteasome condensation under the same stress condition, suggesting the requirement for nucleocytoplasmic shuttling of the proteasome and/or other factors. Moreover, NPC components were detected in cytosolic stress granules after hyperosmotic shock, indicative of disruption of the nuclear pore. It remains to be determined how this happens and whether it underlies the unique localization of proteasome foci at the nuclear envelope.

To understand the nuclear function of p62, Fu et al. knocked out p62 from cells and replaced with a nuclear-trapped version, p62ΔNES [108]. Under basal conditions, p62ΔNES spontaneously underwent LLPS, and the resulting nuclear droplets contained not only ubiquitinated proteins (K48- and K63-linked) but also E1, E2, E3 enzymes, active 26S proteasomes and DUBs, representing all components of the UPS. Indeed, p62ΔNES promoted proteasomal degradation of several nuclear substrates, including NLS-GFP-CL1, free proteasome subunits and the transcription factors c-myc and c-jun. Interestingly, these proteasome-containing p62 condensates could fuse with sucrose-induced proteasome condensates [106]. Compared to p62-null cells, p62ΔNES-reconstituted cells showed better survival after oxidative stress and heat shock, pointing to a cytoprotective role of p62 via regulating nuclear PQC [108].

Another form of stress is nutrient deprivation. In yeast, carbon (glucose) starvation leads to PSG formation, while nitrogen starvation promotes proteasome degradation by autophagy (“proteaphagy”) [30,31,114,115]. On the contrary, Uriarte et al. found that nitrogen (amino acid) starvation of mammalian cells induced nuclear proteasome condensates that they named SIPAN (Starvation-Induced Proteasome Assemblies in the Nucleus) [109]. Again, this structure contained active 26S proteasomes, K48-linked ubiquitin chains, Rad23B, as well as PSME3/REGγ. Ubiquitination and Rad23B, but not PSME3, were required for SIPAN formation, whereas detergent and hypotonic treatments quickened its dissipation. Interestingly, non-essential amino acids (NEAA), but not essential amino acids (EAA), could effectively block SIPAN formation and promote its resolution. SIPAN resolution also depended on the DUB activity of UCH37/UCHL5 and USP14 but was independent of E1, p97/VCP or proteasome activity when nutrients were available. Unlike the observations made by Yasuda et al. with hyperosmotic stress [106], amino acid deprivation did not cause nucleolar stress. Instead, prolonged amino acid starvation triggered p53-mediated apoptosis, while depletion of Rad23B (which eliminated SIPAN) or PSME3 inhibited upregulation of p53 and its target genes, maintaining cell survival under starvation. Although a clear link between these pro-apoptotic factors and SIPAN has yet to be established, the investigators noticed an inverse correlation between SIPAN formation and cell survival upon starvation. Compared to non-cancerous cells, cancer cells and oncogene-transformed “normal” cells showed a much reduced propensity to form SIPAN when deprived of amino acids. In this sense, SIPAN differs from all the other nuclear proteasome condensates discussed above and appears to have a deleterious effect on cells, which was suggested to be a potential defense mechanism against cancer [109].

In all, various stress conditions have been shown to trigger condensation of nuclear proteasomes via LLPS. These structures can be viewed as distinct entities based on the signals that regulate their formation/dissipation, their biochemical compositions and biological functions, but they may also be related both biochemically and functionally. Table 1 summarizes the commonalities and distinctions between these proteasome condensates.

## 3. Proteasomes at the Membranes

**Figure 1:**
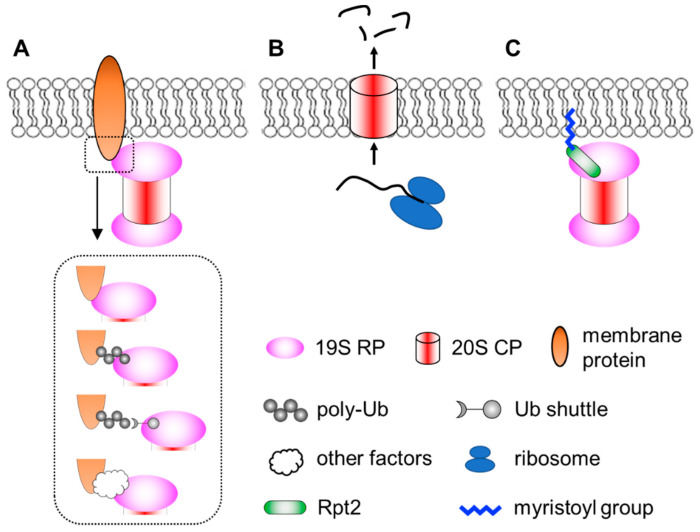
A simplified view of different modes of proteasome–membrane interaction. (**A**) In most cases, proteasomes attach to the membrane by directly or indirectly binding to resident membrane proteins, which may be modified by ubiquitination. (**B**) Neuronal membrane proteasomes (NMP) can degrade nascent, unfolded polypeptides. (**C**) Proteasomes tethered to the membrane via N-myristoylated Rpt2, which is evolutionarily conserved from yeast to human.

Membrane localization of the proteasome has been documented since the early 1990′s [116,117]. Numerous subsequent studies have documented proteasomes in close contact with nuclear envelope-ER [92,118,119,120,121,122,123,124], the Golgi apparatus [125,126], endosomes [127,128], plasma membrane [129,130], mitochondria [123,131,132,133,134,135,136] and so on. Proteasomes at the membranes are particularly important for organelle quality control processes, such as ER-associated degradation (ERAD), endosome and Golgi-associated degradation (EGAD) and mitophagy [120,125,126,137,138,139,140,141]. In addition, proteasomes located at neuronal synapses are also critical for neurotransmission and synaptic plasticity [142,143,144,145,146]. In these cases, the proteasome associates peripherally with the membrane by binding to membrane-resident proteins. This can occur directly between proteasome subunits and membrane proteins. Alternatively, proteasomes can be indirectly recruited to the membrane through binding to the ubiquitin moiety of modified membrane proteins, proteasome-interacting proteins (sometimes in concert with motor proteins and cytoskeleton) [147,148] or even RNAs that function as protein scaffolds [149]. Together, these represent the most common mode of proteasome–membrane interaction, while the proteasome can also locate to the membrane in two other ways, as elaborated below (Figure 1).

### 3.1. Neuronal Membrane Proteasomes

Ramachandran et al. reported a surprising type of membrane-associated proteasomes designated as the neuronal membrane proteasomes (NMPs) [150,151]. As the name suggests, these proteasomes are found at pre- and post-synaptic plasma membranes in neurons, which were confirmed by immunogold electron microscopy (IEM), surface biotinylation, immunofluorescence imaging with antibody feeding and proteinase protection assays. NMPs are thought to be comprised of the 20S CP only, since no 19S components (such as Rpt5 or Rpn1) were found by IEM in these particular membrane proteasomes. NMPs are capable of degrading newly synthesized polypeptides, which are still unfolded, to short peptides. More fascinatingly, the authors showed that these peptide products could exit the cells through NMPs and be released into the synaptic cleft to function as neurotransmitters. Therefore, NMPs function not only as a protein degrader but also a new form of membrane channel to mediate cell–cell communications [152]. Although these findings were very unique and intriguing, the molecular and biochemical details of the NMPs remain unclear. First, it is curious that the 20S CP, which is soluble and hydrophilic, could be fully embedded within the hydrophobic membrane. How is the CP targeted to the plasma membrane and how does it overcome the energy barrier to traverse the lipid bilayer? It was proposed that glycoproteins, such as GPM6, could facilitate this process [151], but a clear mechanistic explanation is still needed. Second, does the NMP exhibit any substrate selectivity? The proposed role of NMPs in cleaving nascent proteins suggests that substrate availability depends on localized protein synthesis by ribosomes in the vicinity [150]. However, if the NMP complex also contained auxiliary factors yet to be identified, it might recognize and process folded protein substrates as well. On the other hand, the recent discovery that the 20S CP can by itself degrade ubiquitinated proteins [153] also implies that NMPs may have a broader range of substrates. A following question is the molecular composition and regulatory mechanisms of the NMPs. Finally, what is the function of NMP in vivo? Additionally, how can we specifically maneuver it for research and therapeutic purposes without affecting the bulk of proteasomes inside the cell? Answering these questions will depend on new technical advances in imaging, chemical biology, proteomics, structural biology and genetic models, which makes it challenging but also rewarding at the same time.

### 3.2. Membrane Targeting of Proteasomes by N-Myristoylation

A third means of targeting the proteasome to the membrane is through lipid modification. N-myristoylation of the Rpt2 subunit has been observed by mass spectrometry in multiple species, ranging from yeast to plants to mammals [154,155,156,157,158,159,160,161]. Typically, N-myristoylation occurs co-translationally on nascent polypeptides still bound to the ribosome, where the 14-carbon saturated fatty acyl group is covalently linked to the second amino acid (almost always a Gly) after the initiator methionine is removed by methionyl aminopeptidase [162,163,164]. Notably, among all proteasome subunits of mammalian cells, Rpt2 is the only one that begins with Met-Gly, serving as the only site of the entire proteasome complex for N-myristoylation. This MG sequence of Rpt2 is strictly conserved from yeast to human, suggesting that Rpt2 is likely to be myristoylated in all species. In yeast, myristoylated Rpt2 has been shown to target proteasomes to the nuclear envelope, which is required for nuclear protein quality control [156,157]. Blocking this modification with the Rpt2-ΔG or Rpt2-G2A mutations causes mislocalization of nuclear proteasomes to the cytosol.

The role of Rpt2 myristoylation in higher organisms has not been rigorously investigated, despite Rpt2 being one of the most abundantly myristoylated proteins in human cells [161]. Our recent work demonstrated that wild-type human Rpt2 proficient for myristoylation was found at the plasma membrane, with some distribution at membrane-bound organelles as well. Membrane localization was abolished by the same ΔG/G2A mutations of human Rpt2. However, in stark contrast with results from yeast, loss of Rpt2 myristoylation in mammalian cells led to Rpt2 enrichment in the nucleus [84]. A serendipitous finding was that myristoylation-mediated membrane association is a prerequisite for Rpt2 phosphorylation at Tyr439 (Y439) by the tyrosine kinase Src, which itself is a well-established myristoylated protein tethered to the membrane [84,165]. Moreover, Rpt2-Y439 phosphorylation could be reversed by the phosphotyrosine phosphatase PTPN2 (also known as T cell PTP or TC-PTP). PTPN2 has multiple splicing isoforms. Rpt2-pY439 could only be dephosphorylated by the membrane-bound isoform of PTPN2 known as TC48, but not by the nuclear isoform TC45 [84]. Hence, the kinase, phosphatase and substrate are all placed in the same neighborhood confined by the membrane.

The biochemical consequence of Rpt2-Y439 phosphorylation is readily conceivable, as it is the very tyrosine residue within the highly conserved HbYX tail (hydrophobic residue—Tyr—any amino acid) of Rpt2 required for RP–CP association. Rpt2-Y439 is the most frequently detected pTyr site of all 19S subunits. The phosphorylation was seen in the developing rat brain but more evidently detected in cancer cells with hyperactive Src [84]. Src-mediated Rpt2-Y439 phosphorylation selectively inhibited the activity of membrane-associated proteasomes as demonstrated by a membrane-targeted reporter protein, Myr^Rpt2^-GFPodc. On the contrary, the Src-specific inhibitor saracatinib/AZD0530 blocked Y439 phosphorylation and enhanced proteasomal degradation of membrane-bound substrates. Importantly, this seemed to be an integral part of the anti-cancer effects of saracatinib, since cancer cells expressing the nonphosphorylatable Y439F mutant were more resistant to this drug, both in vitro and in vivo [84]. Thus, reversible phosphorylation of Rpt2-Y439 provides a unique example of localized regulation of membrane-associated proteasomes.

## 4. Conclusions and Future Perspectives

Proteasome localization is highly dynamic within the cell and may be remarkably heterogeneous between cell types. This is an important basis of compartmentalized protein degradation that is widely conserved through evolution. Nonetheless, we have seen differences between yeast and mammalian cells where the behavior and fate of the proteasome are differentially controlled by specific factors. We are just beginning to get in-depth understanding of intracellular proteasome targeting and trafficking in higher organisms, and it remains a daunting mission to obtain a complete picture of localized function and regulation of the proteasome across different cell types, species and growth/stress conditions. A yet more challenging task would be to confirm these findings in vivo and to develop new tools for “site-specific” manipulation of proteasomes at any particular location in a cell.

A prerequisite for achieving these goals is a deeper and better characterization of proteasome composition, modification, interactome and its microenvironment within a cell. Researchers have been empowered by state-of-the-art techniques, including proximity labeling (e.g., BioID/TurboID, APEX, PUP-IT) [166,167,168], quantitative proteomics, super-resolution imaging and cryo-electron tomography (cryo-ET) [21,22,106,120,122] to probe and catalog the contents of proteasome-containing subcellular structures. Chemical biology approaches involving metabolic labeling, click chemistry, genetic code expansion and cross-linking mass spectrometry (XL-MS) have provided critical insights into proteasome modification and assembly [83,161,169,170,171]. Commonly used reporter proteins (e.g., GFPu, GFPodc, Ub^G76V^-GFP, UBL-CP8-35) can be engineered to reflect local proteasome activity at defined compartments [84,108,172,173], while knock-in mice bearing fluorescence protein-tagged proteasome subunits would be valuable to monitor proteasome distribution and dynamics in vivo [174]. With classic yeast genetics and CRISPR screens, many more regulators of the proteasome are expected to be uncovered [93,114].

Finally, some further possibilities may be speculated. 1. In addition to the above discussed, what other mechanisms may be used for proteasome targeting? Can we alter proteasome localization (and function) pharmacologically, optically, mechanically, magnetically or acoustically [175]? Does lipid modification (i.e., N-myristoylation) promote exosomal secretion of the proteasome, as has been shown with palmitoylated ACE2 [176]? Can we design “Proteasome-TACs” that recruit proteasomes directly to the substrates (or vice versa) for therapeutic use? A recently discovered small circular RNA seemed to do precisely that [149]. Along this line, since proteasome subunits have been identified as RNA-binding proteins [177,178], can RNAs act as molecular tethers between the proteasome and chromatin or other proteins [179]? 2. As mentioned earlier, cancer cells show reduced SIPAN formation [109], and tyrosine phosphorylation of membrane-bound Rpt2 is relevant to the anti-cancer effect of saracatinib [84]. This makes one wonder whether proteasome (mis)localization can be considered as a biomarker for disease diagnosis and treatment. Moreover, it is unclear whether subcellular localization of the proteasome is altered in multiple myeloma patients after proteasome inhibitor treatment, or in patients with proteasome-associated autoinflammatory syndrome (PRAAS) who carry congenital mutations in proteasome genes [180,181]. Relocation of the proteasome may cause changes to the local proteome and rewire intracellular signaling, which may lead to a different kind of proteasome-oriented therapy.

## Figures and Tables

**Table 1:** Characteristics of nuclear proteasome condensates under various stress conditions.

Reference	Yasuda et al. [106]	Lee et al. [107]	Fu et al. [108]	Uriarte et al. [109]
Condensate induced by	Hyperosmotic stress	Hyperosmotic stress	Nuclear retention of p62, oxidative and heat stress	Nutrient starvation (NEAA depletion)
Formation depends on	Ubiquitination, Rad23B, UBE3A	Ubiquitination, nucleocytoplasmic trafficking	Ubiquitination, protein synthesis, p62	Ubiquitination, Rad23B
Clearance depends on	Proteasome activity, p97/VCP, UCH37/UCHL5	Proteasome activity	Proteasome activity	UCH37/UCHL5, USP14
Driven by	LLPS	LLPS	LLPS	LLPS
Form of proteasome	Active, 26S holoenzyme	Active, 26S holoenzyme	Active, 26S holoenzyme	Active, 26S holoenzyme
Substrates of proteasome	Orphan ribosome proteins (RPs)		NLS-GFP-CL1, unassembled proteasome subunits, c-myc, c-jun	
Other UPS-related components	Ub chains (K48-linked but not K63-linked, K11/K48), UCH37 [111]	Ub chains (K48-linked)	Ub chains (K48-linked and K63-linked) E1/E2/E3s, DUBs, chaperones	Ub chains (K48-linked)
Accompanied phenotypes	Nucleolar stress	Condensates near NE, Nups found in stress granule	p62 condensates can fuse with those induced by sucrose [106]	No nucleolar stress. Cells protected by NEAA but not EAA
Biological function	Prevent RP aggregation, protect cells from hyperosmotic stress	Protect cells from hyperosmotic stress	Nuclear PQC. Protect cells from heat stress	A possible defense mechanism against oncogenic transformation

## Data Availability

Not applicable.
